# Interpretable Quantification of Scene-Induced Driver Visual Load: Linking Eye-Tracking Behavior to Road Scene Features via SHAP Analysis

**DOI:** 10.3390/jemr18050040

**Published:** 2025-09-09

**Authors:** Jie Ni, Yifu Shao, Yiwen Guo, Yongqi Gu

**Affiliations:** 1 School of Automotive and Traffic Engineering, Jiangsu University, Zhenjiang 212013, China; yfshao1215@163.com (Y.S.); 18720885687@163.com (Y.G.); gyq3319524341@163.com (Y.G.); 2 Changzhou Engineering and Technology Institute, Jiangsu University, Zhenjiang 213000, China

**Keywords:** urban scenario, road traffic safety, urban design, human factors, driving visual load, visual attention demand, explainable AI, SHAP analysis

## Abstract

Road traffic accidents remain a major global public health concern, where complex urban driving environments significantly elevate drivers’ visual load and accident risks. Unlike existing research that adopts a macro perspective by considering multiple factors such as the driver, vehicle, and road, this study focuses on the driver’s visual load, a key safety factor, and its direct source—the driver’s visual environment. We have developed an interpretable framework combining computer vision and machine learning to quantify how road scene features influence oculomotor behavior and scene-induced visual load, establishing a complete and interpretable link between scene features, eye movement behavior, and visual load. Using the DR(eye)VE dataset, visual attention demand is established through occlusion experiments and confirmed to correlate with eye-tracking metrics. K-means clustering is applied to classify visual load levels based on discriminative oculomotor features, while semantic segmentation extracts quantifiable road scene features such as the Green Visibility Index, Sky Visibility Index and Street Canyon Enclosure. Among multiple machine learning models (Random Forest, Ada-Boost, XGBoost, and SVM), XGBoost demonstrates optimal performance in visual load detection. SHAP analysis reveals critical thresholds: the probability of high visual load increases when pole density exceeds 0.08%, signage surpasses 0.55%, or buildings account for more than 14%; while blink duration/rate decrease when street enclosure exceeds 38% or road congestion goes beyond 25%, indicating elevated visual load. The proposed framework provides actionable insights for urban design and driver assistance systems, advancing traffic safety through data-driven optimization of road environments.

## 1. Introduction

Urban traffic accidents remain a critical public health issue, and improving urban road safety requires multifaceted approaches. Researchers in urban planning and management suggest that urban landscapes and built environments are closely related to traffic safety, as drivers’ perception and feedback on their driving environment affect the overall safety of urban traffic [1,2]. Kaygisiz et al. (2017) discussed the impact of road alignment, building coverage, lane count, land use types, and the number of bus stops on urban traffic safety [3]. Asadi et al. (2022) analyzed the relationship between multiscale built environment factors—such as the year of housing construction, land use type, road hierarchy, cycling activities and infrastructure, proximity characteristics, and neighboring variables—and the number of traffic accidents [4]. With advancements in street view maps and the rapid development of deep learning in computer vision, discovering the quantitative relationship between urban landscape elements and traffic safety has become an effective method for assessing urban traffic safety, providing a solid basis for improving the urban road environment [5,6,7,8]. However, current studies often rely on subjective assessments [9] or historical traffic accident data [10,11,12], and there is insufficient research on the mechanisms by which urban environmental elements influence drivers and consequently contribute to traffic accidents.

Scholars in the field of driver assistance systems aim to improve traffic safety by studying the impact of driving scenarios on drivers’ physiological load and attention. Compared to highways and rural roads, urban environments are often more complex. Variables such as intersections, dynamic changes at bus stops, pedestrians and non-motorized vehicles, parked vehicles, traffic signs, and billboards significantly increase the amount of visual information drivers need to process [13], thereby increasing their driving load. Visual load is a critical factor affecting drivers’ ability to process visual information [14]. Sustained high-intensity driving loads can lead to driver fatigue, delayed reactions, more frequent operational errors, and poor decision-making, all of which impair driving performance and are a major cause of urban traffic accidents [15,16,17,18]. Therefore, by monitoring drivers’ physiological indicators, facial expressions, and eye movements, constructing multimodal feature fusion models to identify high-load states and provide warnings can effectively reduce the risk of traffic accidents [19,20,21]. Nevertheless, while existing approaches can monitor visual load through physiological signals, they rarely deconstruct the combined effects of road scene elements or provide actionable optimization suggestions for urban design.

Building upon these findings, this study proposes an interpretable analytical framework that quantifies scene-induced visual load by establishing correlations between eye-tracking behavior and road scene features through SHAP analysis. Specifically, the research will: (1) classify visual load levels using eye movement indicators; (2) identify high-impact urban scene elements and their dynamic effects on visual load; and (3) elucidate the mechanisms through which scene modifications influence eye movements and consequently alter visual load. This approach is expected to provide a novel data-driven paradigm for assessing urban traffic safety based on scene-visual load interactions.

## 2. Literature Review

### 2.1. Review of the Impact of Urban Landscape and Built Environment on Traffic Safety

In recent years, with the rapid pace of urbanization and advancements in computer vision technology, the impact of urban scene elements and built environments on traffic safety has become a research hotspot. Using technologies such as remote sensing and street view images, researchers have aimed to uncover the complex relationships between road environments, driving behavior, and traffic safety. Liu et al. (2023), utilizing high-resolution remote sensing and street view images combined with deep learning-based semantic segmentation techniques, analyzed the visual landscape features of urban expressways [1]. Their results established a model linking road landscape features with driver fatigue, showing that landscape richness can reduce fatigue and improve traffic safety. Similarly, Cai et al. (2022) applied deep neural networks to extract visual environment features from Google Street View images to study the impact of these visual features on speeding-related accidents [6]. They found that tree coverage along roadsides was linked to fewer speeding accidents, while highly complex visual environments increased collision risks.

In the study of built environments and pedestrian safety, González et al. (2023) combined collision prediction with built environment detection using panoramic street view images [22]. They showed that built environment elements strongly influence pedestrian collision risks. Bustos et al. (2021) proposed an automated method that used classification, segmentation, and explainability techniques to capture dangerous urban landscapes [23]. Their study found that reducing the space between roads and buildings while increasing green open spaces could improve pedestrian safety. Additionally, Tanprasert et al. (2022) identified road accident hotspots with street view image recognition and semantic segmentation techniques [24]. They discovered that the size and proximity of surrounding objects were key factors influencing accident susceptibility. Qin et al. (2020), using graph convolutional networks, analyzed the relationship between urban road networks and traffic congestion [25]. They found that physical, functional, and topological features of the built environment are critical in shaping congestion, offering strategies to prevent jams and enhance road safety.

In summary, existing literature has demonstrated that urban scene semantics and built environment features profoundly affect driver behavior and traffic safety. Factors such as the complexity of road environments and the layout of greenery can significantly impact driver performance, accident rates, and pedestrian safety.

### 2.2. Review of Scenarios and Driving Visual Load

Different driving scenarios require drivers to process varying degrees of visual information, which is a key factor influencing driving visual load. By studying the characteristics of these scenarios, we can gain a deeper understanding of the sources of driving load and optimize driving conditions to reduce driver strain. Foy et al. (2018) designed four road types in a simulated environment with different scene elements [26]. They found that when the road environment shifted from a simple two-lane road with a median strip to the more complex “city center” road, drivers’ psychological cognitive load increased significantly. Liu et al. (2011) used a driving simulator where drivers wore eye-tracking devices to examine the effects of varying information levels on highway signage in a visual recognition experiment [27]. They reported that when a sign contained more than five elements, drivers’ eye movement patterns changed markedly, and recognition effectiveness declined. He et al. (2017) utilized a non-invasive eye-tracking system to record drivers’ eye movement parameters as they drove through highway tunnels under two lighting conditions [28]. They showed that reduced brightness, lower uniformity, and flicker inside tunnels enlarged pupil diameter and increased visual load. Ahlström et al. (2018) studied two different driving scenarios: a winding, low-traffic rural environment and a suburban road with higher traffic density and denser roadside environments, finding that driver demands and visual load varied between the two [29]. Fiolić et al. (2023) investigated the relationship between road markings and signs with different visibility levels in nighttime conditions and driver behavior and cognitive load [30]. They discovered that improved visibility positively impacted cognitive load, with optimal cognitive load occurring under high visibility. These studies, while providing partial insights into the impact of environmental factors on visual workload, predominantly focus either on isolated scene elements or generalized scenario comparisons. They fail to deliver either comprehensive quantitative analysis of all elements within specific driving contexts or establish an interpretable correlation model connecting scene features, oculomotor behavior, and visual load dynamics.

### 2.3. Review of Driver Attention Studies

When drivers face excessive attention demands, the driving visual load correspondingly increases, which not only heightens psychological and physiological stress but may also negatively impact driving safety. The study by Cvahte et al. (2019) [31] points out that visual and cognitive attention demands directly affect drivers’ ability to perceive changes in the traffic environment. When drivers’ attention is distracted, they are more prone to making driving errors, thereby increasing accident risk. Gaze occlusion, eye-tracking, and physiological measurements are three effective methods for assessing driver attention. Gaze occlusion can identify when and for how long drivers need to observe the traffic environment, while also estimating residual attention. Kircher et al. (2020) found that attention demands on highways increase as following distance decreases and traffic density rises, which reduced occlusion frequency and duration [32]. Liu et al. (2021) set up urban road traffic scenarios involving multiple traffic variables and conducted gaze occlusion experiments with 30 participants to explore the traffic factors that influence attention demands in urban environments [13].

In the field of eye-tracking, Zeng et al. (2023) examined prolonged driving through simulation and eye-tracking [33]. They found that longer driving reduced fixation durations but increased saccade amplitudes, indicating impaired visual sensitivity and risk assessment. Regarding physiological indicators, Aminosharieh et al. (2023) used EEG, ECG, and EDA signals in a simulated environment to assess drivers’ attention states [34]. Liu et al. (2023) further confirmed through EEG studies that brain activity differs significantly under varying cognitive loads, particularly in high-cognitive-load tasks where drivers’ attention is more easily disrupted [35]. Given the limitations of quantifying driving visual load through physiological indicators, this study introduces visual attention demand as an indicator to explore the correlation between drivers’ visual metrics and attention demands. Based on this, driving visual load is quantified, aiming to provide a more reasonable and reliable method for predicting and evaluating driving visual load.

## 3. Methodology

### 3.1. Methodological Framework

**Figure 1:**
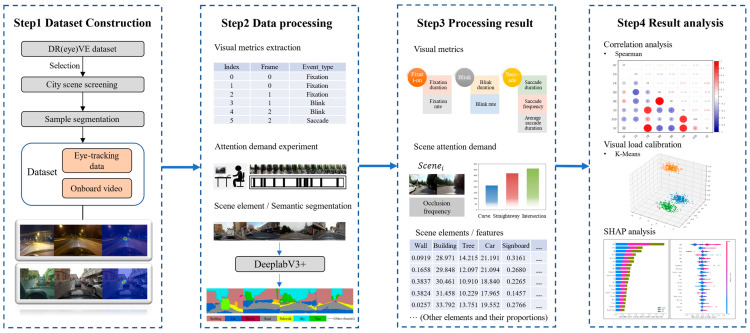
Overall methodological framework.

The overall framework of this study is illustrated in Figure 1, in which * is used as a locator identifier. First, using the DR(eye)VE dataset [36], we filtered and selected scenes related to urban environments, extracted the corresponding in-vehicle video and eye-tracking data, and segmented the samples to construct the urban driving dataset for this research.

Next, based on the eye movement event types identified from raw eye-tracking data, we calculated the corresponding visual metrics. For each scene sample, we extracted fixation metrics (fixation duration and fixation rate), blink metrics (blink duration and blink rate), and saccade metrics (saccade duration, saccade frequency, and average saccade amplitude). For the in-vehicle video data, we conducted a standardized visual occlusion experiment to quantify attention demand: the driver’s attention demand in each scene sample was characterized by the frequency of visual occlusion operations performed by the experimenter, covering typical urban scenarios such as curves, straight roads, and intersections. Using a pre-trained DeeplabV3+ semantic segmentation pipeline, we extracted scene element information from each video frame, calculating pixel-wise proportions of key elements (e.g., vehicles, pedestrians, traffic signs) to represent environmental characteristics. Frame-level features were then averaged to generate scene-level representations.

Finally, Pearson correlation analysis was performed to examine the relationship between visual metrics and attention demand, thereby identifying eye movement features significantly associated with scene-induced visual load. Based on these significantly correlated visual metrics, K-means clustering was applied. By integrating the distribution of attention demand intensity with spatial mapping of cluster centroids, we achieved quantitative calibration of visual load levels. Following calibration, interpretable predictive modeling was implemented: machine learning models were constructed to simultaneously predict visual load levels and eye movement metrics from scene features. SHAP value analysis was then conducted to quantify the directional effects of specific scene features on visual load and eye movement behavior, providing an actionable decision-making basis for optimizing urban road environments to reduce visual load.

### 3.2. Data Source

In this study, we use the DR(eye)VE dataset as the basis and filter the portions related to urban driving scenarios, including driving videos captured in urban environments at 1080 p/25 fps resolution by a GARMIN VirbX camera mounted on the roof of the vehicle, and the raw eye-tracking data of the driver collected by an SMI ETG 2w commercial eye-tracking glasses. These are used to construct the urban driving dataset. The eye-tracking device contains two built-in cameras dedicated to tracking the pupil at 60 fps, providing information on the driver’s gaze, including fixation, saccadic movements, and blinks. We aim to combine the driver’s eye-tracking data with the onboard camera video to analyze the driver’s visual attention demand and driving visual load in complex urban environments. A total of 800 urban driving scenes were selected from the dataset as analysis samples, each lasting 5 s. The raw data consists of urban driving videos recorded by the onboard camera and corresponding driver eye movement parameters.

### 3.3. Attention Demand Experiment

Driving attention demand refers to the amount of traffic information a driver needs to process per unit of time or distance [37]. The vision occlusion technique can be used to assess when and where a driver needs time to gather traffic information [38]. The default state of vision occlusion can be either occluded or non-occluded; the duration of occlusion can be controlled by the driver or set by the experiment. Some scholars have set the default state of vision occlusion as non-occluded, allowing the driver to choose when to occlude their vision and for how long, to study the characteristics of attention demand during high-speed driving [39]. Vision occlusion provides an effective method for studying attention demand in different driving environments by simulating restricted vision. By analyzing drivers’ occlusion behavior in various scenes, we can better understand their attention demand and driving visual load.

**Figure 2:**
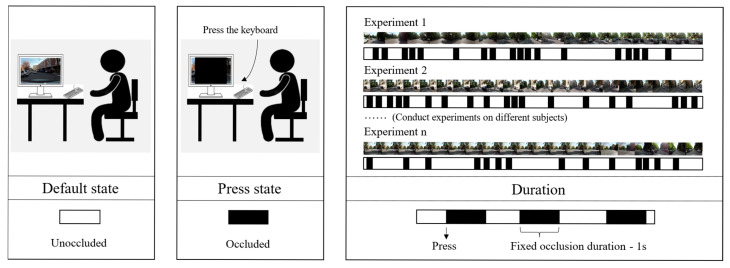
Visual occlusion experiment.

As shown in Figure 2, urban driving videos were played on a monitor, and the occlusion of the forward view was controlled by a keyboard in this study. When the participant felt they no longer needed to observe the traffic environment, they pressed the space bar to occlude the view ahead, simulating the driver’s gaze moving away from the road. The occluding rectangle in the forward view automatically disappeared after a period of time.

Some studies indicate that with longer durations of occlusion, occlusion behavior tends to concentrate on specific, highly complex segments of the route, whereas brief, 1 s occlusions promote a more uniform distribution of behavior across the entire experimental route [40]. Additionally, related driving simulator experiments found that under a 1 s occlusion condition, vehicle longitudinal and lateral control performance was satisfactory and more closely resembled actual driving conditions; in contrast, when the occlusion duration reached 2 s, driver uncertainty increased significantly [41]. Consequently, to achieve a more uniform distribution of occlusion behavior across the test course, avoid the systematic bias introduced by extended occlusion periods clustered in a few highly complex segments, and preserve ecological validity, the occlusion duration was fixed at 1 s. All experimental scenes came from the DR(eye)VE dataset, labeled as urban driving scenes. We collected the frame positions corresponding to when the participant pressed the space bar to match the different samples in the urban driving dataset established in this study.

In addition, to approximate real-world driving conditions as closely as possible, the present study emphasized the following aspects in its experimental design:

(1) Participant selection: All participants held a valid People’s Republic of China motor vehicle driver’s license and possessed extensive driving experience, ensuring sufficient familiarity with and responsiveness to urban traffic environments.
(2) Visual environment simulation: A laboratory display of appropriate size was carefully selected for the driving video presentation, with the aim of reproducing drivers’ natural visual environment as realistically as possible.
(3) Driving state induction: Prior to the formal experiment, a non-experimental urban driving video was shown to help participants gradually adapt to the simulated driving environment. During the experiment, the laboratory was kept quiet and lighting was dimmed to facilitate immersion in the driving task.
(4) Task logic aligned with reality: The occlusion task was designed to be actively triggered by participants, thereby simulating the self-initiated gaze shifts that occur in real driving and better reflecting the natural mechanisms of attentional allocation.
(5) Although certain differences between laboratory conditions and real-world driving are unavoidable, the above design measures ensured that, under controlled conditions, the visual task characteristics and decision-making processes of urban driving were reproduced to the greatest extent possible. This provided a solid experimental foundation for the quantification of attentional demands.

Given that the occlusion duration was fixed, we consider that more frequent occlusions in a driving scene indicate more remaining attention, reflecting a lower attention demand. Therefore, in this study, we calculated the occlusion frequency (the number of occlusions per sample divided by the sample duration) for different urban driving samples to inversely represent the driver’s attention demand in various urban driving scenes.

### 3.4. Visual Metrics Extraction

In urban driving scenarios, attention demand and driving visual load are key factors affecting driving safety and efficiency. High driving visual load typically results in changes in visual metrics, such as increased fixation duration and fixation rate, altered blink rate, and changes in saccade frequency.

There is a strong correlation between visual metrics such as fixation, saccade, and blink, and attention demand and driving visual load. Fixation duration and fixation rate are important metrics for evaluating the driver’s attention to critical information in the environment. When the driver encounters complex traffic situations or needs to process a large amount of information, fixation duration and fixation rate typically increase to better capture and process the information [42,43]. On the other hand, saccade duration and saccade frequency reflect the driver’s efficiency in visual search and environment scanning. Under high driving visual load conditions, the saccade duration and saccade frequency may increase, indicating that the driver requires more time to process information [44,45].

Blink duration and blink rate are also effective indicators of attention and fatigue. In high-load driving environments, blink rate and blink duration may decrease, indicating changes in the driver’s attention under stress or fatigue [46,47].

In summary, changes in eye movement metrics can reflect the driver’s attention demand and driving visual load, serving as an important tool for assessing driving visual load [48]. This provides a significant reference for designing driving safety and assistance systems. Therefore, in this study, we selected fixation metrics (fixation duration, fixation rate), saccade metrics (saccade duration, average saccade duration, saccade frequency), and blink metrics (blink duration, blink rate) to analyze the correlation between visual metrics and attention demand (driving visual load).

The DR(eye)VE dataset contains driver eye movement parameters collected by eye-tracking equipment with a sampling frequency of 60 fps, continuously recording the driver’s gaze points and eye movement event types (including fixation, saccade, and blink). Based on the raw eye-tracking data from the dataset, we developed a program to calculate seven eye movement metric characteristics for each sample. Fixation duration is the number of fixation events divided by the sampling frequency of the eye tracker, and fixation rate is the number of fixations per sample divided by the total sample time. Saccade duration is the number of saccade events divided by the sampling frequency of the eye tracker, average saccade duration is the total saccade duration divided by the number of saccades in the sample, and saccade frequency is the number of saccades per sample divided by the total sample time. Blink duration is the number of blink events divided by the sampling frequency, and blink rate is the number of blinks per sample divided by the total sample time. The results of these calculations were used for subsequent correlation analysis between visual metrics and attention demand (driving visual load), as well as for calibrating the driver’s attention demand level (or driving visual load level).

### 3.5. Scene Features Extraction

#### 3.5.1. Semantic Segmentation Model

**Figure 3:**
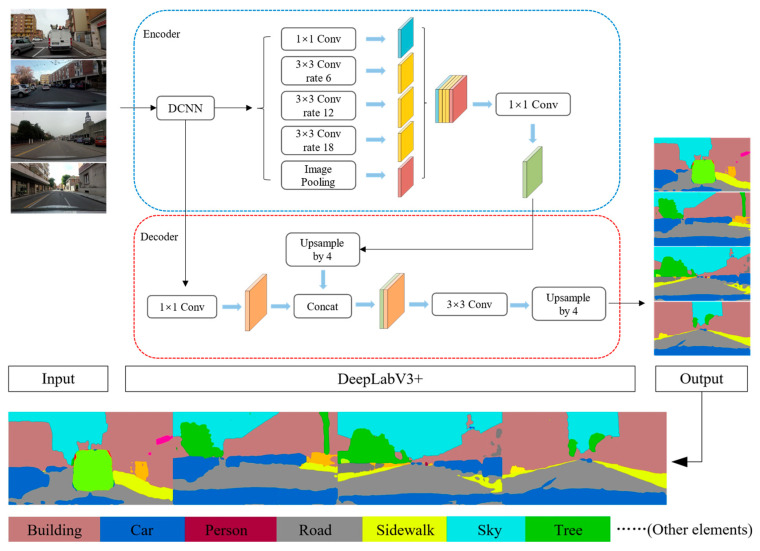
Semantic segmentation extracts scene features.

Most of the external environmental information during driving is obtained visually by the driver [49]. In this study, we use the Deeplabv3+ model, pre-trained on the ADE20K dataset, from the MMsegmentation module in OpenMMLab [50] to extract semantic information from environmental images to represent the driving environment. Each pixel of the image after semantic segmentation is assigned to a specific element category and marked with a different color. The ADE20K dataset contains 150 categories, as shown in Figure 3. The pre-trained Deeplabv3+ model can recognize elements including roads, trees, and the sky, covering almost all scene elements in urban driving environments.

#### 3.5.2. Element Characteristics Extraction

The urban driving scene videos in the dataset are processed by extracting each frame, which is then input into the pre-trained Deeplabv3+ model for semantic segmentation. After the semantic segmentation is completed, the proportion of each scene element in each frame is calculated. The proportion of scene elements is the ratio of the pixels of a specific element to the total pixels of the scene. The formula is as follows:

(1) $${frame\_prop}_{ij}=\frac{\sum_{k=1}^{n}{pixel}_{ik}}{\sum_{i=1}^{150}\sum_{k=1}^{n}{pixel}_{ik}}\;\;\;\;\;\;\;$$

where ${frame\_prop}_{ij}$ represents the proportion of the *i*-th scene element in the *j*-th frame (i = 1, 2, …, 150), and ${pixel}_{ik}$ refers to the *k*-th pixel of the *i*-th element.

Once the scene element proportions for each frame are calculated, the average of the proportions for all frames in a scene sample is taken to represent the scene element characteristics. The formula is as follows:

(2) $$\;\;{scene\_prop}_{i}=\;\frac{\sum_{j}^{m}{frame\_prop}_{ij}}{m}$$

where ${scene\_prop}_{i}$ is the average proportion of the *i*-th scene element in the scene sample (*i* = 1, 2, …, 150), and *m* is the total number of frames in the sample.

After extracting the scene element features through semantic segmentation for all scene samples in this study, we found that 30 elements, such as walls, buildings, and trees, accounted for more than 99% of the total. Thus, these 30 elements are considered sufficient to represent the urban driving environment. Therefore, the proportions of these 30 scene elements were selected to quantify the urban driving environment characteristics, which were used as feature variables for subsequent analysis of urban driving visual load based on attention demand.

In addition, based on the results of scene semantic segmentation, this study further established the Green Visibility Index (GVI), Sky Visibility Index (SVI), and Road Congestion (RC), among other indicators, to further analyze the element characteristics of urban driving scenes.

The GVI reflects the visibility of urban vegetation, which has a positive effect on the driver’s psychological comfort and relaxation. The SVI is an important indicator for evaluating the esthetic quality of the urban environment, which may affect the driver’s environmental cognition and processing. The RC directly affects driving behavior and decision-making processes, while Road Width (RW) is related to speed control and driving safety. The Street Canyon Enclosure (SCE) and Building View Index (BVI) are closely related to the driver’s environmental perception and sense of security. The Traffic Mix (TM) affects drivers’ cognitive processing of the environment, thereby influencing their driving behavior and decision-making processes, which is related to driving safety.

This study particularly focuses on the above scene features to analyze their impact on driving visual load and visual metrics based on attention demand, with the calculation formulas of the indicators shown in Table 1.

### 3.6. Machine Learning Methods

#### 3.6.1. Clustering Algorithm for Driving Visual Load Calibration

In this study, the K-Means clustering algorithm was used to calibrate the driver’s driving visual load levels based on attention demand. K-Means is a widely used unsupervised learning algorithm that partitions data points into K clusters by minimizing the distance between the data points and the cluster centroids. Before applying K-Means, the selected visual metrics were standardized to eliminate the influence of different dimensions and magnitudes. The optimal number of clusters was determined using the silhouette coefficient and DBI index. The clustering results helped identify patterns of different driving visual load levels, reflecting the driver’s attention demand under various driving scenarios.

After completing the K-Means clustering, we preliminarily calibrated the driving visual load levels for each group based on the occlusion frequency. We further analyzed the centroid values of each group, which represent the center of the cluster in the visual metric feature space. To validate the rationality of the driving visual load level calibration after clustering, we combined the correlation analysis results between visual metrics and occlusion frequency. This verified whether the visual metric changes in each group’s centroids followed a consistent trend (positive correlation if driving visual load levels increased with higher visual metrics, negative correlation otherwise). Ultimately, the driving visual load levels for each group were determined.

#### 3.6.2. Predictive Modeling for Visual Load and Eye Movement Metrics

In this study, Random Forest (RF), Adaptive Boosting (AdaBoost), eXtreme Gradient Boosting (XGBoost), and Support Vector Machine (SVM) were used to construct detection models for driving visual load and visual metrics. These algorithms are well-suited for handling complex datasets and capturing non-linear relationships. These four algorithms were chosen for their proven performance in classification and regression tasks, with each employing different principles. RF, AdaBoost, and XGBoost are ensemble algorithms based on decision trees, with RF using a bagging strategy and the latter two using boosting strategies [51,52,53]. Additionally, SVM was included for comparison as it classifies by finding the maximum-margin hyperplane.

The driving visual load level and driver’s eye movement metrics were taken as outputs, and the feature variables of urban driving scene extracted by semantic segmentation technology were taken as inputs to train various machine learning models, so as to seek the influence relationship between scene features, driving visual load and driver’s eye movement metrics.

#### 3.6.3. SHAP-Based Interpretability Analysis

SHAP (SHapley Additive exPlanations) is a method for explaining machine learning model outputs. Based on the concept of Shapley values from cooperative game theory, it quantifies each feature’s contribution to model predictions. SHAP was introduced by Lundberg and Lee in 2017 [54], with the primary goal of providing a clear and accurate explanation for each prediction, revealing the contribution of individual features to the model’s output.

The Shapley value, derived from cooperative game theory, measures each participant’s contribution to the total value in a cooperative setting. In machine learning, this concept is applied to features, calculating each feature’s contribution to the model’s predictions. For a given prediction

*f*(*x*) and feature vector *x*, the SHAP value $\phi_{i}$ provides a contribution metric for each feature $x_{i}$, calculated as:

(3) $$\phi_{i}\;=\;\sum_{S\;\subseteq \;N\;\setminus \;\{i\}}\frac{\left|S\right|!\left(\left|N\right|\;-\;\left|S\right|\;-\;1\right)!}{\left|N\right|!}\;\left[\;f\left(S\cup \;\{i\}\right)\;-\;f\left(S\right)\;\right]$$

where *N* is the set of all features, *S* is a subset of the feature set *N* excluding $x_{i}$, and *f*(*S*) is the model’s prediction using the feature subset *S*. The absolute values of $\left|S\right|$ and $\left|N\right|$ represent the sizes of subset S and the universal set of features N, respectively.

The formula represents the weighted average of the marginal contribution of feature $x_{i}$ to the prediction across all possible subsets of features. In this study, SHAP values are used to explain the impact of each feature on the classification labels in the classification models.

## 4. Results

### 4.1. Attention Demand and Visual Metrics

#### 4.1.1. Correlation Analysis

Using various visual metrics as independent variables and occlusion frequency across different scenarios as the dependent variable, a Spearman correlation analysis was conducted to examine the relationship between visual metrics and the driver’s attention demand (or driving visual load). Significant visual metrics were selected as a basis for establishing driving visual load levels based on attention demand.

The results are shown in Table 2, five metrics related to blinking and saccades—blink duration, blink rate, saccade duration, average saccade duration, and saccade frequency—are significantly associated with attention demand, while fixation duration and fixation rate were not. Saccade duration, saccade frequency, and average saccade duration were positively correlated with attention demand: higher values for these metrics corresponded to higher attention demand and higher driving visual load. Conversely, blink rate and blink duration were negatively correlated with attention demand: lower blink rate and shorter blink duration indicated higher attention demand and driving visual load.

In previous driving research, fixation duration and fixation rate have often been employed as key indicators for assessing driving load and attentional allocation [43,55]. However, the sensitivity of these measures varies across tasks and experimental paradigms. On the one hand, prolonged fixations may not necessarily reflect increased information-processing demands from the external environment, but rather internal, non-driving-related cognitive activities [56]. On the other hand, prior studies have suggested that dynamic measures such as blinks and saccades exhibit greater sensitivity to changes in cognitive load in complex scenarios [57]. Furthermore, given that the scenarios in this study primarily involved multi-lane urban roads and intersections, drivers were required to frequently switch their gaze across multiple areas of interest (rear-view mirrors, side windows, pedestrians, traffic signals). Such frequent shifts often interrupted fixations, leading to numerous short fixations or mixed scan–fixation patterns. This, in turn, may have reduced the sensitivity of conventional fixation-based metrics (duration/rate) to overall attentional demands, resulting in the lack of a significant association between fixation-related measures and attention demand in this study.

#### 4.1.2. K-Means Clustering Based on Visual Metrics

From the results of the correlation analysis, five visual metrics—blink duration, blink rate, saccade duration, average saccade duration, and saccade frequency—were significantly correlated with attention demand. These five metrics were used to determine the driver’s driving visual load levels based on attention demand.

**Figure 4:**
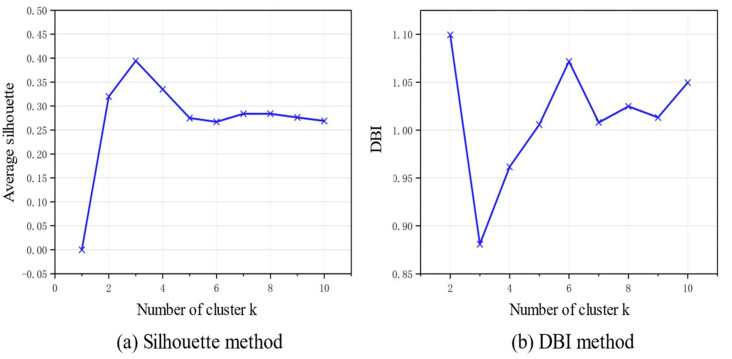
Clustering performance evaluation. A larger silhouette coefficient and a smaller DBI (Davies–Bouldin Index) indicate greater separation between clusters and higher compactness within clusters, thereby reflecting better clustering performance.

First, the eye movement metrics were standardized using the z-score method. Then, the K-Means clustering algorithm was applied to label the driving visual load levels. The number of clusters was set from 1 to 10, and the clustering results were evaluated using the silhouette coefficient and DBI index. As shown in Figure 4, it was found that when the number of clusters was 3, the silhouette coefficient was the highest (0.394) and the DBI value was the lowest (0.881).

Moreover, according to Wickens’ Multiple Resource Theory (MRT) model [58], increased workload does not invariably lead to performance decline. Both excessively high and excessively low workload levels can impair performance. Excessive workload may cause errors, difficulties, or performance deterioration, whereas sustained underload may result in boredom, loss of situational awareness, and reduced vigilance. This reflects the three workload states of underload, optimal load, and overload. Furthermore, studies such as Huang et al. (2024) have successfully employed an N-back task in simulated driving to construct a three-level workload classification model (low, medium, high), thereby validating the effectiveness of a three-class structure for identifying drivers’ mental workload [59]. Accordingly, the present study adopts a three-class framework to categorize drivers’ visual workload states, which is theoretically grounded and statistically justified.

After clustering, the average occlusion frequencies of the three groups were 0.3352, 0.2805, and 0.2132, respectively. Variance analysis on occlusion frequencies across the groups revealed an F-value of 6.155 and a *p*-value of 0.002 (*p* < 0.05), indicating significant differences between groups. Thus, the groups were labeled as low-load, medium-load, and high-load based on attention demand.

Additionally, in Table 3, combining the correlation analysis results of visual metrics and attention demand (where “↓” indicates a negative correlation and “↑” indicates a positive correlation) with the centroids of the clusters, it was observed that the average visual load states of the groups were consistent with the relationships between metrics and attention demand, confirming the rationality of this classification.

In conclusion, the three clustered groups were labeled as low-load, medium-load, and high-load, with respective sample sizes of 148, 438, and 214. The results were included in Table 4.

### 4.2. Driving Visual Load and Scene Features

#### 4.2.1. Construction and Evaluation of the Visual Load Detection Model

With the driving visual load levels as labels, 35 characteristics variables (excluding the proportions of the sky and road elements, as they were represented by SVI and RW characteristics) were used as inputs. The dataset was randomly divided into training and testing sets in an 8:2 ratio. Considering the imbalance between low, medium, and high load samples, Synthetic Minority Oversampling Technique (SMOTE) was employed to rebalance the training set. SMOTE generates synthetic samples to bridge the gap between minority and majority classes, resulting in a more even distribution in the feature space, which improves the model’s performance in identifying minority classes.

Five-fold cross-validation was implemented to train four models based on different methods, aiming to prevent overfitting and ensure the model’s reliability in detecting driving load in new datasets. Grid search combined with five-fold cross-validation was used to traverse all combinations of key hyperparameters to identify the optimal configuration for the best driving load detection model.

The models were evaluated using precision, recall, and F1-score, as summarized in Table 5. It was observed that the Random Forest model and the XGBoost model achieved the best performance on the original and balanced datasets, respectively. Moreover, all four models performed better on the balanced datasets than on the original datasets, confirming that using SMOTE can effectively alleviate the issue of data imbalance and improve classification performance.

Given that the Random Forest model performed best on the original dataset and exhibited good performance on the balanced dataset, the trained Random Forest model was used for subsequent SHAP analysis to examine the influence of different scene features on driving visual load.

#### 4.2.2. Effect Analysis of Scene Features on Drivers’ Visual Load

**Figure 5:**
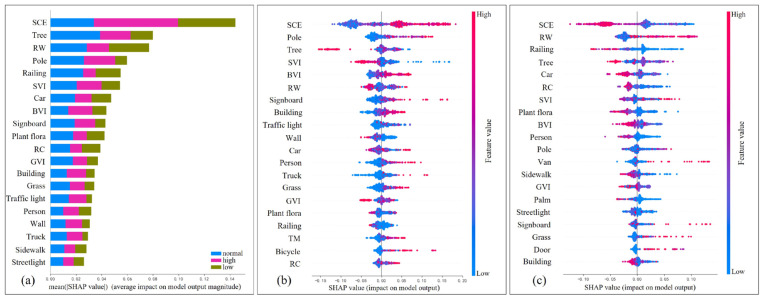
Ranking of the importance of scene features on visual load. (**a**) Overall ranking of SHAP value importance for scene features influencing low/medium/high visual workload states; (**b**) ranking of SHAP value importance for scene features influencing the high visual workload state; (**c**) ranking of SHAP value importance for scene features influencing the low visual workload state. In the figure, the color of each point represents the magnitude of the feature value, while its position indicates the SHAP value of the feature for the corresponding load state. A positive SHAP value indicates that the feature increases the probability of that load state occurring, whereas a negative SHAP value indicates a decreased probability.

Figure 5a shows the SHAP importance rankings of all features, with the top ten characteristics being SCE, tree proportion, RW, pole proportion, railing proportion, SVI, car proportion, BVI, signboard proportion, and plant flora proportion. The results indicate that these urban driving environment characteristics have a significant impact on the driving visual load state.

Since high load conditions may increase driver fatigue, potentially leading to traffic accidents and reduced road safety, maintaining a low load condition for drivers becomes a key objective. Therefore, further SHAP importance rankings for high and low load states were plotted and analyzed, as shown in Figure 5b,c.

As shown in Figure 5b, in high-load states, static elements such as poles, trees, signboards, and buildings have the most significant influence. The proportion of poles, signboards, and buildings is positively correlated with the probability of a high-load state, meaning that the larger the feature value, the higher the likelihood of the high-load state occurring. In contrast, the proportion of trees is negatively correlated with the probability of a high-load state, so a higher feature value corresponds to a lower probability of a high-load state. Among dynamic elements, cars and people have the most significant impact. The proportion of people is positively correlated with the probability of a high-load state, with a higher feature value leading to a greater likelihood of the high-load state occurring. The relationship between car proportions and high-load states is more complex. Among comprehensive scene indicators, SCE and SVI have the most significant impact on the occurrence of high-load states. The SCE is positively correlated with the probability of a high-load state, meaning the higher the index, the more likely a high-load state will occur. On the contrary, SVI is negatively correlated with the probability of a high-load state, with higher values corresponding to a reduced likelihood of a high-load state.

As shown in Figure 5c, in low-load states, static elements such as railings, trees, and plant flora have the most significant influence. The proportions of railings and plant flora are negatively correlated with the probability of a low-load state, meaning that as their values increase, the probability of a low-load state decreases. The relationship between tree proportions and low-load states is more complex. Among dynamic elements, cars and people still have the most significant impact. The proportions of both cars and people are negatively correlated with the probability of a low-load state, with higher feature values leading to a decreased probability of a high-load state. Among comprehensive scene indicators, SCE, RW, and road RC have the most significant influence on the occurrence of low-load states. The SCE and RC are negatively correlated with the probability of a low-load state, meaning that as their values increase, the likelihood of a low-load state decreases. However, RW is positively correlated with the probability of a low-load state, with larger values leading to an increased likelihood of a low-load state.

**Figure 6:**
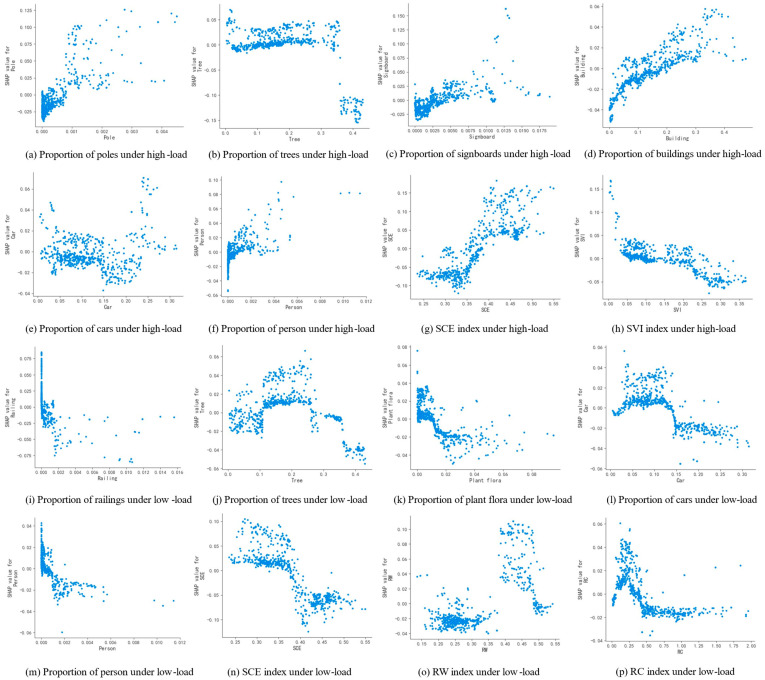
SHAP dependence plots of key scene features under high and low load states. The *X*-axis represents the value range of the corresponding feature, and the *Y*-axis represents the SHAP value of that feature, indicating the change in probability it contributes to the model output.

To further analyze how different scenes affect drivers in both high and low visual states, this study generated SHAP dependence plots. These plots visually show how changes in feature values affect the driver’s visual load state, providing a quantitative analysis of the feature’s influence:

(1) Among static elements, as shown in Figure 6a, when the proportion of poles exceeds 0.08%, the probability of the driver being in a high-load state increase. In urban environments, elements such as traffic sign poles, traffic signal poles, and utility poles are categorized as pole elements. The increase in pole elements signifies a rise in environmental complexity. More poles may indicate additional traffic signs or indicative boards, which draw the driver’s attention and elevate attention demand, thereby increasing visual load. As shown in Figure 6b,j, tree elements have different effects on the driver’s high-load and low-load states. When the proportion of trees exceeds 35%, the probability of the driver being in a high-load state significantly decreases. This may be because trees create a more relaxing environment and simplify the visual field, thus reducing attention demand and alleviating visual load. In contrast, under low-load conditions, the impact of tree elements is more complex. When the proportion of trees is between 11% and 26%, the probability of a low-load state increases. However, when tree coverage exceeds 26%, the probability decreases, showing an initial rise followed by a decline. This suggests that a moderate proportion of trees reduces visual load, while proportions that are too low or too high increase complexity and raise attention demand. As shown in Figure 6c,d, when the proportion of signboard and building elements exceeds 0.55% and 14%, respectively, the probability of a high-load state increases. Signboard elements, which include signs and billboards, tend to distract drivers. Therefore, an excess of signboards in the scene heightens attention demand. Similarly, a high concentration of buildings suggests a more complex driving environment, thereby increasing driver attention demand and consequently raising visual load. As shown in Figure 6i,k, the increase in railing and plant flora elements both decrease the probability of the driver being in a low-load state. Railings are typically located in road repair areas, accident-prone zones, or places where boundaries need to be emphasized. These conditions require constant awareness, thereby increasing attention demand and visual load. When the proportion of plant flora exceeds 1.2%, it may cause additional distractions, thereby somewhat increasing the driving visual load.
(2) Among dynamic elements, car and person have the most significant impact on the driving visual load, which is an evident result. As shown in Figure 6e,l, as the proportion of cars increases, the probability of a high-load state initially decreases. However, when the proportion reaches 24%, the probability of a high-load state rises sharply, with SHAP values turning positive. This indicates that an excessive number of cars in the scene increases the driver’s attention demand, leading to a rise in driving load. This is also confirmed under the low-load condition: when the proportion of cars exceeds 13%, the probability of the driver being in a low-load state drops suddenly, triggering an increase in driving load. As the most unpredictable factor in the traffic system, person has an even more pronounced impact on driver load. When pedestrians appear in the scene, the probability of a high-load state rises, while the probability of a low-load state decreases. This shows that pedestrians add instability to the environment. Drivers must monitor not only the road ahead but also the behavior of roadside pedestrians to prevent accidents. Such demands increase attention and raise driving visual load. As dynamic elements in urban driving environments, car and person significantly influence the driver’s distraction and attention. Ensuring traffic safety thus requires effective management of vehicles and pedestrians within the traffic system.

(3) Among comprehensive scene indicators, as shown in Figure 6g,n, SCE has the most significant impact on both high-load and low-load visual states. When the SCE exceeds 39%, the probability of a high-load state increases, and when it exceeds 36%, the probability of a low-load state decrease. A higher SCE indicates a more spatially enclosed environment, which narrows the driver’s field of view and raises environmental complexity. This adds distracting elements, makes it harder for drivers to perceive their surroundings, and increases attention demand, thereby raising visual load. As shown in Figure 6h, under high-load conditions, when SVI reaches 16%, the probability of the driver being in a high-load state decreases. A higher SVI suggests a wider field of view with fewer elements in the forward vision, reducing complexity and lowering attention demand. This helps relieve the driver’s high-load state. As shown in Figure 6o, when RW reaches 38%, the probability of a low-load state suddenly increases. An increase in RW implies a higher proportion of road elements, which means fewer attention-demanding elements such as vehicles and pedestrians. It also provides a broader field of view, reducing environmental complexity and thus lowering the driver’s load. Additionally, RC is another important factor affecting driver load. As shown in Figure 6p, when RC reaches 40%, the probability of a low-load state decrease. A higher RC reflects more vehicles on the road. An excessive number of dynamic elements increases cognitive load, raises attention demands, and consequently elevates driving visual load.

### 4.3. Visual Metrics and Scene Features

#### 4.3.1. Model Construction for Analysis

Based on the correlation analysis in Section 4.1.1, the driver’s visual metrics, including blink metrics (blink duration and blink rate) and saccade metrics (saccade duration, average time per saccade, and saccade frequency), show a certain degree of correlation with the driver’s attention demand. Therefore, this study selects these five visual metrics as dependent variables and the 35 extracted scene feature variables as independent variables to analyze how driving scenes affect changes in visual metrics and further influence the driver’s load.

In this study, five different machine learning regression models were trained based on the five visual metrics. All samples were randomly divided into training and test sets in an 8:2 ratio. Additionally, 5-fold cross-validation and grid search were used to traverse all combinations of key hyperparameters across the four models to determine the optimal hyperparameter combination, thereby obtaining the best regression models.

Among the five models, the XGBoost method consistently achieved the best performance, with $R^{2}$ values of 0.94, 0.85, 0.75, 0.73, and 0.78, respectively. Thus, the trained XGBoost models were used in the subsequent SHAP analysis to evaluate the influence of different scene characteristics on visual metrics.

#### 4.3.2. Effect Analysis of Scene Features on Driver’s Eye Movement Behavior

(1) Blink Metrics

**Figure 7:**
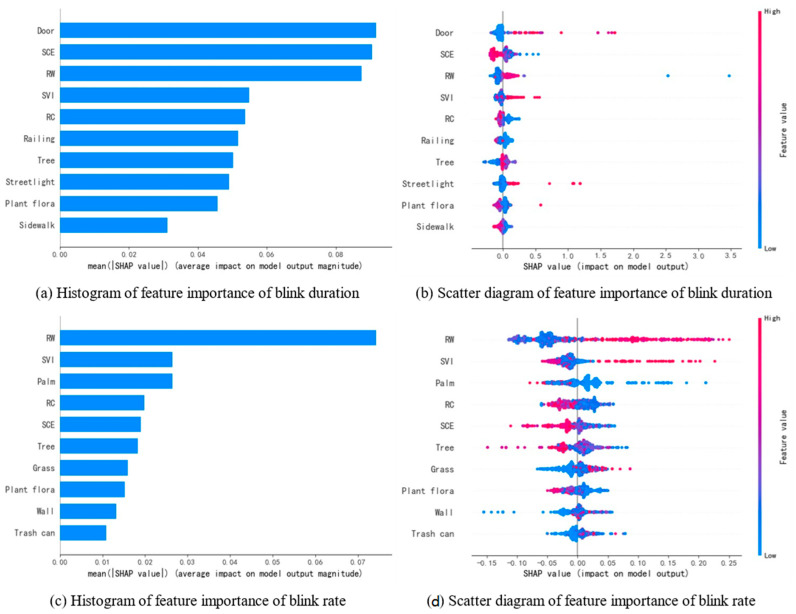
Ranking of the importance of scene features on blink-related indicators. (**a**,**c**) show the SHAP value importance ranking of scene features influencing blink duration and blink rate, respectively; (**b**,**d**) present SHAP feature density scatter plots of scene features influencing blink duration and blink rate. In the scatter plots, the color of each point represents the magnitude of the feature value, while its position indicates the SHAP value of the feature for the corresponding indicator. A positive SHAP value indicates that the feature increases the indicator value, whereas a negative SHAP value indicates a decrease.

Figure 7a,c list the ten scene characteristics that have the greatest influence on blink duration and blink rate, respectively. Among them, door, SCE, RW, SVI, and RC have the greatest impact on blink duration, while RW, SVI, palm, RC, and SCE have the greatest impact on blink rate. It can be observed that the four comprehensive indicators—SCE, RW, SVI, and RC—have a more significant effect on blink metrics compared to other simple scene feature ratios. Therefore, this study further plots SHAP dependence diagrams for these four indicators to analyze their impact on blink metrics.

**Figure 8:**
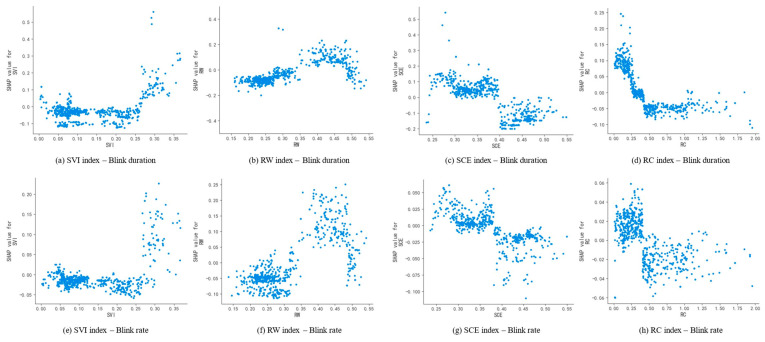
SHAP dependence plots of key scene features for blink duration and blink rate. The *X*-axis represents the value range of the corresponding feature, and the *Y*-axis represents the SHAP value of that feature, indicating the magnitude of change it contributes to the model output.

As blink duration and blink rate are both blink-related metrics, scene characteristics have similar effects on them. As shown in Figure 8a,e, when SVI exceeds 25%, the SHAP value rises and becomes greater than zero, leading to an increase in blink duration and blink rate. According to the previous correlation analysis, blink metrics are negatively correlated with the driver’s attention demand, suggesting that a high level of SVI may reduce the driver’s attention demand, thus positively contributing to reducing driving load. As shown in Figure 8b,f, when RW is between 35% and 47%, the SHAP value is greater than zero, indicating that too narrow or too wide a road width decreases blink duration and blink rate, thereby increasing the driver’s load. As shown in Figure 8c,g, when SCE reaches 40%, the SHAP value for blink duration turns from positive to negative, and similarly, when SCE reaches 38%, the SHAP value for blink rate also turns from positive to negative. This indicates that a high level of SCE increases the complexity of the scene and the driving pressure, requiring the driver to focus more on acquiring environmental information, thereby reducing blink duration and blink rate and increasing driving load. As shown in Figure 8d,h, when RC reaches 25%, the SHAP value for blink duration becomes negative, and it remains negative after reaching 35%. For blink rate, the SHAP value turns from positive to negative when RC reaches 40%. As RC increases, the excessive number of vehicles forces the driver to constantly monitor the movement of other vehicles to ensure driving safety. This increases the time required to gather information, reducing blink duration and blink rate, which leads to an increase in driving visual load.

(2) Saccade Metrics

**Figure 9:**
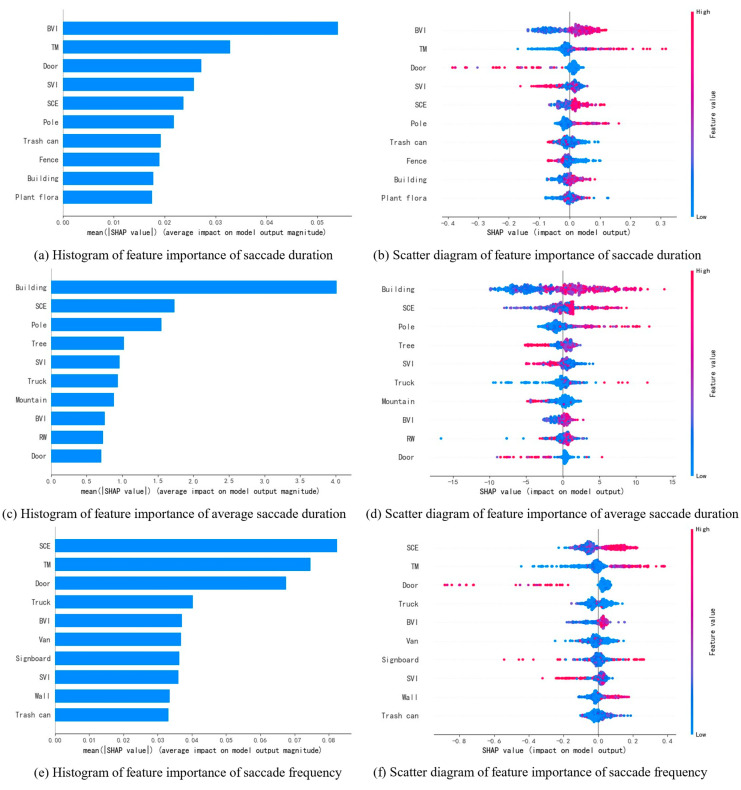
Ranking of the importance of scene features on blink-related indicators. (**a**,**c**,**e**) show the SHAP value importance rankings of scene features influencing saccade duration, average saccade duration and saccade frequency, respectively; (**b**,**d**,**f**) present SHAP feature density scatter plots of scene features influencing saccade duration, average saccade duration and saccade frequency. In the scatter plots, the color of each point represents the magnitude of the feature value, while its position indicates the SHAP value of the feature for the corresponding indicator. A positive SHAP value indicates that the feature increases the indicator value, whereas a negative SHAP value indicates a decrease.

Figure 9a,c,e list the top ten scene characteristics that most significantly affect the driver’s saccade duration, average saccade duration, and saccade frequency, respectively. BVI, TM, door, and SVI have the greatest impact on the driver’s saccade duration; building, SCE, pole, and tree have the greatest influence on the average saccade duration; and SCE, TM, door, and truck have the greatest impact on saccade frequency. SHAP dependency diagrams were plotted for these scene characteristics to provide further analysis.

**Figure 10:**
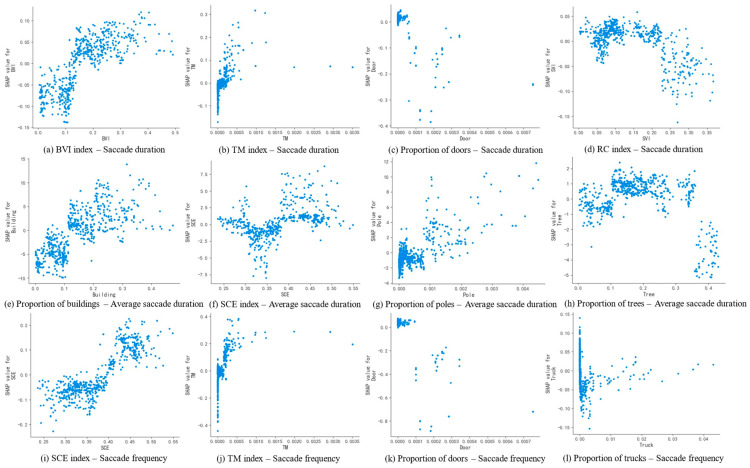
SHAP dependence plots of key scene features for saccade duration, average saccade duration, and saccade frequency. The *X*-axis represents the value range of the corresponding feature, and the *Y*-axis represents the SHAP value of that feature, indicating the magnitude of change it contributes to the model output.

As shown in Figure 10a,b, when BVI and TM reach 13% and 0.02%, respectively, the SHAP values for saccade duration turn positive. High BVI increases the complexity of static elements in the scene, while high TM indicates the presence of more non-motorized vehicles and pedestrians, which undoubtedly adds to the scene’s information load. Since saccadic behavior is the driver’s way of acquiring information from the scene, increased saccade duration results in higher driving load. According to Figure 10c,k, the presence of door elements in the scene at a certain proportion reduces both saccade duration and frequency. A possible explanation for this interesting phenomenon is that when the proportion of door elements is high, the driver’s attention is more likely to be focused on the door elements, reducing the time spent scanning and searching for unrelated information, which in turn decreases saccadic behavior to some extent. As shown in Figure 10d, when RC reaches 20%, the driver’s saccade duration decreases, reducing the complexity of the driving scene and alleviating driving load.

In Figure 10e–g, when the proportions of building, SCE, and the proportions of pole elements reach 11%, 39%, and 0.1%, respectively, the average saccade duration increases. This implies that the driver’s saccadic searches become more detailed and cover a larger area. An increase in building elements likely requires the driver to process more building-related information, increasing the visual complexity of the scene. Meanwhile, high SCE may require the driver to spend more time evaluating spatial relationships and navigation paths. Additionally, although the proportion of pole elements is relatively small, their presence suggests the existence of more signs and other elements that could distract the driver’s visual search, thus increasing the time per saccade and raising driving load. As shown in Figure 10h, when the proportion of tree elements reaches 36%, the SHAP value for the average saccade duration remains negative. This indicates that a higher proportion of tree elements can effectively reduce the driver’s saccadic search time. A possible reason is that a high proportion of tree elements may obscure certain visual elements along the roadside, simplifying the visual scene on either side of the driver’s field of view, thus reducing the time and space required to search the environment and lowering driving load.

According to Figure 10i,j, when SCE and TM reach 40% and 0.02%, respectively, the driver’s saccade frequency increases. Similarly, this is a result of increased complexity in both static and dynamic elements in the scene. High proportions of buildings, facilities, pedestrians, and non-motorized vehicles increase the amount of information in the scene, leading to more frequent visual searches and higher driving load. As shown in Figure 10l, the presence of truck elements decreases the driver’s saccade frequency. This may be because truck elements, being particularly hazardous in the traffic system, are more likely to capture the driver’s attention compared to other elements in the scene, leading to more focused attention and, consequently, a reduction in the driver’s saccadic behavior.

## 5. Discussion

### 5.1. Comparison with Existing Studies

SHAP interpretability analysis revealed that the presence of billboards or signage and increased pedestrian or vehicular traffic elevates drivers’ visual workload, whereas vegetation has a mitigating effect. These findings are consistent with those of White and Shah (2019), who highlighted the restorative characteristics of natural scenes [60]. Additionally, drivers’ visual workload increases with building complexity, which aligns with experimental results reported by Edquist et al. (2012) [61] and Foy et al. (2018). [26]. The SHAP analysis further indicated that a tree coverage proportion of 11–26% favors the occurrence of low workload states, whereas increases in building proportion and street canyon enclosure (SCE) exacerbate drivers’ visual workload. This is similar to Cai’s findings [6], where a certain proportion of trees reduced the number of speeding-related collisions, while increased building proportion and visual complexity were associated with a rise in such incidents—possibly because drivers face difficulties handling heightened visual complexity in emergency situations [62].

In prior road safety research, including studies by Cai, Kaygisiz, and Asadi [3,4,6], the focus has largely been on macro-level factors such as traffic data, road structure, land use attributes, and socio-demographic characteristics to establish their association with traffic accidents. However, the occurrence of accidents is influenced by a complex interplay of factors that extend beyond the driver to the environment and vehicle, making such approaches relatively coarse-grained. In contrast, this study innovatively centers on drivers’ visual workload—a critical safety factor—and emphasizes a detailed analysis and quantitative mapping of its direct source: the visual environment. This perspective not only deepens understanding of the intrinsic mechanisms of driving safety but also provides a novel pathway for human-factor-based interventions in traffic safety.

Moreover, existing studies have primarily focused on the independent associations between road environment features and driving performance [61] or eye movement behavior [63]. Some studies have applied eye-tracking technology to detect driving workload [64,65], but these largely remain at the binary “feature–behavior” or “feature–workload” level. In contrast, this study employs SHAP analysis to not only reveal the mechanisms through which urban driving environment features influence drivers’ visual workload, but also further elucidate their effects on various eye movement parameters, a key mediating factor. This establishes a systematic scene–eye–workload explanatory chain, overcoming the limitations of previous studies that insufficiently captured the dynamic human-factor response process.

### 5.2. Response to Research Objectives

First, this study employed a visual occlusion method to quantify visual attention demands across different urban scenarios, confirming a significant correlation between eye-movement indicators and visual attention requirements. Based on this, the K-Means clustering algorithm was applied to classify drivers’ visual load.

Subsequently, using machine learning techniques combined with SHAP interpretability analysis, the study identified key elements of urban scenes and their dynamic effects on visual workload. For example, when street canyon enclosure (SCE) exceeded 39%, the probability of drivers being in a high-load state increased significantly, whereas when it exceeded 36%, the probability of a low-load state decreased markedly. Moreover, SHAP analysis further elucidated how different scene features influence the mediating changes in drivers’ eye-movement indicators, revealing the mechanism through which urban scenes affect visual load. For instance, excessively high SCE reduces blink metrics and increases saccade metrics, thereby resulting in higher visual load.

### 5.3. Practical Implications

Based on SHAP-based interpretations of how specific urban scene features affect drivers’ visual workload and eye-movement behavior, this study translates its analytical findings into actionable strategies for urban planners and traffic safety policymakers. These measures aim to reasonably alleviate visual workload and enhance road safety:

(1) Regulation of street canyon visual complexity

The study found that excessively high street canyon enclosure (SCE) strongly influences reduced blink indicators and increased saccade indicators. When SCE exceeds 39%, the probability of high workload states rises significantly. Therefore, planning and design in high-risk urban areas (e.g., intersections, ramps) should consider controlling SCE. Measures may include establishing building setback regulations, managing street block height-to-width ratios, and introducing periodic visual-open buffer zones to break continuous enclosure and maintain sky visibility.

(2) Visual optimization of green landscapes

SHAP analysis results showed that tree coverage between 11–26% facilitates low workload states, whereas coverage exceeding 35% significantly reduces the probability of high workload. Accordingly, targeted roadside greening programs should be implemented in urban streetscape design. In areas with high SCE (e.g., dense urban canyons), tree planting should be prioritized to leverage its workload-reducing effects, while in open areas, tree density should be kept below 26% to avoid adding visual complexity and to maintain visual comfort.

(3) Management of visual interference from street facilities

The presence of poles (>0.08%) and billboards (>0.55%) significantly increases the probability of high visual workload. Thus, optimizing the layout and density of street furniture is necessary to reduce visual clutter. Specifically, planning regulations can limit unnecessary billboards, encourage the use of large integrated signs instead of multiple small ones, and in high-workload areas, consider overhead gantry structures as alternatives to roadside poles to minimize horizontal line-of-sight interference.

(4) Optimization of visual openness and road geometry

Sky view index (SVI > 16%) and roadway width (RW > 38%) are significantly associated with reduced visual workload. Where feasible, restrictions on overhead structures and the adoption of stepped building designs in narrow blocks should be encouraged to enhance sky visibility. During design and planning, particularly for segments with high cognitive demand, maintaining or increasing roadway width above 38% can provide a broader field of view and reduce feelings of spatial confinement.

(5) Dynamic traffic management strategies

Road congestion (RC > 40%) and the presence of pedestrians and vehicles—particularly when vehicle density exceeds 24%—substantially increase workload. Intelligent traffic management systems should be deployed to monitor traffic density in real time. When congestion exceeds critical thresholds, variable message signs can be used to recommend alternative routes. Signal timing optimization can reduce vehicle–pedestrian conflicts, and in high pedestrian-density areas, pedestrianization or segregated pedestrian systems should be considered to minimize sudden visual disturbances for drivers.

### 5.4. Limitations and Future Directions

Despite the extensive scope of this study, several limitations remain:

(1) The quantification of scene features primarily relied on pixel-based measures, which did not fully capture geometric attributes such as line structures and shapes, dynamic motion information, or semantic elements such as road markings, thus limiting the richness of feature representation.
(2) Assessment of drivers’ visual workload was primarily based on eye-movement indicators, lacking integration with multimodal physiological data (e.g., EEG, ECG), which constrains the comprehensive reflection of drivers’ cognitive and physiological states.
(3) This study focused on urban driving environments from the DR(eye)VE dataset, covering typical routes in several European cities. The homogeneity in road types, traffic culture, and climatic conditions limits the generalizability of the model to rural, highway, or other regional contexts, and the model’s applicability in non-urban environments (e.g., highways, rural roads, or extreme weather conditions) remains unverified.

For future research, feature extraction can be enhanced by incorporating advanced computer vision models, such as attention-based semantic segmentation networks or graph neural networks, to integrate geometric, dynamic, and contextual semantic information, thereby providing a more comprehensive representation of driving scenes. In terms of workload assessment, multimodal physiological signals (e.g., EEG, ECG, galvanic skin response) should be integrated to construct a more robust and holistic evaluation system for drivers’ visual workload. Regarding model generalization, large-scale validation across diverse road types and cross-cultural driving scenarios is necessary, alongside exploration of domain adaptation and transfer learning techniques, to improve the model’s applicability and reliability in complex real-world environments.

## 6. Conclusions

During driving, the visual environment serves as the primary source of drivers’ visual load and is a key basis for predicting it. Drivers’ eye movement parameters effectively reflect their visual load levels, and different scene elements exert distinct influences on visual load and eye-movement behavior. By employing SHAP analysis, these effects can be clearly elucidated, enabling the construction of a systematic explanatory pathway linking “scene–eye movements–load.” This study not only provides a scientific method and tools for understanding drivers’ visual load in urban driving environments, but also offers targeted strategies and recommendations for urban planning and traffic management, thereby reducing visual load and enhancing road safety.

## Figures and Tables

**Table 1:** Comprehensive features of urban driving scenes.

Index	Implication
Green Visibility Index (GVI)	The ratio of the number of vegetation pixels to the total number of pixels in the image
Sky Visibility Index (SVI)	The ratio of the number of sky pixels to the total number of pixels in the image
Road Width (RW)	The ratio of the number of road pixels to the total number of pixels in the image
Street Canyon Enclosure (SCE)	The ratio of the number of pixels of buildings, vegetation, traffic signs, fences, walls, and poles to the total number of pixels in the image
Building View Index (BVI)	The ratio of the number of building pixels to the total number of pixels in the image
Road Congestion (RC)	The ratio of the number of vehicle pixels to the number of road pixels
Traffic Mix (TM)	The ratio of the number of non-motor vehicle and pedestrian pixels to the number of motor vehicle pixels

**Table 2:** Spearman correlation analysis of drivers’ visual metrics and attention demand.

Visual Index	Fixation Duration (s)	Fixation Rate (Times/s)	Blink Duration (s)	Blink Rate (Times/s)	Saccade Duration (s)	Average Saccade Duration (ms)	Saccade Frequency (Times/s)
Correlation coefficient (*p*-value)	−0.019 (0.849)	−0.186 (0.064)	0.239 (0.017 *)	0.262 (0.008 **)	−0.272 (0.006 **)	−0.357 (0.000 **)	−0.234 (0.019 *)

Note: ** and * represent 1% and 5% significance levels, respectively.

**Table 3:** Calibration of cluster centroid value and driving load class.

Centroid of Each Group	Blink Duration (↓)	Blink Rate (↓)	Saccade Duration (↑)	Average Saccade Duration (↑)	Saccade Frequency (↑)	Tentative Load Status
**Group 1**	1.4853	1.5881	−0.9653	−0.1188	−1.2978	Low-load
**Group 2**	−0.3322	−0.3350	−0.3318	−0.4998	0.0354	Medium-load
**Group 3**	−0.3406	−0.3998	1.1687	0.9378	0.7384	High-load

**Note**: “↓” indicates that a lower value corresponds to a higher load; “↑” indicates that a higher value corresponds to a higher load.

**Table 4:** Driving visual load classification results.

Group	Load Condition	Sample Size	Proportion
**Group 1**	Low-load	148	18.50%
**Group 2**	Medium-load	438	54.75%
**Group 3**	High-load	214	26.75%

**Table 5:** Model performance evaluation.

Index		Original Dataset				Balanced Dataset			
		RF	XGB	Ada	SVM	RF	XGB	Ada	SVM
Precision	Low-load	86.41	79.82	78.50	81.25	93.60	95.69	94.04	93.77
	Medium-load	91.98	88.62	90.00	84.48	92.13	94.69	92.96	91.64
	High-load	86.40	87.09	83.65	87.57	87.15	88.86	82.46	88.44
	Macro average	88.26	85.18	84.05	84.43	90.96	93.08	89.82	91.28
	Weighted average	87.99	86.27	84.57	85.61	90.93	93.06	89.90	91.25
Recall	Low-load	80.91	79.09	76.36	82.73	93.03	94.24	90.91	95.76
	Medium-load	81.87	81.32	79.12	80.77	90.11	93.13	87.09	90.38
	High-load	93.10	91.09	89.66	89.08	89.66	91.67	90.52	87.93
	Macro average	85.29	83.83	81.71	84.19	90.93	93.01	89.50	91.36
	Weighted average	87.81	86.25	84.38	85.63	90.88	92.99	89.44	91.27
F1-Score	Low-load	83.57	79.45	77.42	81.98	93.31	94.96	92.45	88.44
	Medium-load	86.63	84.81	84.21	82.58	91.11	93.91	89.93	91.01
	High-load	89.63	89.04	86.55	88.32	88.39	90.24	86.30	88.18
	Macro average	86.61	84.44	82.73	84.30	90.94	93.04	89.56	91.32
	Weighted average	87.73	86.19	84.31	85.60	90.90	93.02	89.52	91.25

## Data Availability

The dataset used in this study is publicly available from the DR(eye)VE Project at http://imagelab.ing.unimore.it/dreyeve (accessed on 11 April 2024) and was originally introduced in Palazzi et al. (2019) [36].
